# Increased FGF-21 Improves Ectopic Lipid Deposition in the Liver and Skeletal Muscle

**DOI:** 10.3390/nu16091254

**Published:** 2024-04-23

**Authors:** Ying Jia, Huixin Yu, Jia Liang, Qiang Zhang, Jiawei Sun, Hongqing Yang, Haijing Yan, Shuping Zhang, Yana Li, Yongjun Jin, Meizi Yang

**Affiliations:** 1 Department of Pharmacology, Binzhou Medical University, Yantai 264003, China; jiaying505@126.com (Y.J.); a17860505823@163.com (H.Y.); jialiang0121@126.com (J.L.); zhangziqiang0304@163.com (Q.Z.); hjyan211@163.com (H.Y.); zsp861122@126.com (S.Z.); 2 College of Basic Medicine, Binzhou Medical University, Yantai 264003, China; sunjiawei09@163.com (J.S.); yanghongqing1101@163.com (H.Y.); 3 Department of Pathophysiology, Binzhou Medical University, Yantai 264003, China; liyanuo@bzmc.edu.cn; 4 Department of Endocrinology, Binzhou Medical University, Yantai 264003, China; endojin@bzmc.edu.cn

**Keywords:** ectopic lipid deposition, FGF-21, PLIN2, PLIN5, FAT/CD36, CPT-1

## Abstract

Obesity can lead to excessive lipid accumulation in non-adipose tissues, such as the liver and skeletal muscles, leading to ectopic lipid deposition and damaging target organ function through lipotoxicity. FGF-21 is a key factor in regulating lipid metabolism, so we aim to explore whether FGF-21 is involved in improving ectopic lipid deposition. We observed the characteristics of ectopic lipid deposition in the liver and skeletal muscles of obesity-resistant mice, detected the expression of FGF-21 and perilipin, and found that obesity-resistant mice showed a decrease in ectopic lipid deposition in the liver and skeletal muscles and increased expression of FGF-21. After inhibiting the expression of FGF-21, a more severe lipid deposition in liver cells and skeletal muscle cells was found. The results indicate that inhibiting FGF-21 can exacerbate ectopic lipid deposition via regulating lipid droplet synthesis and decomposition, as well as free fatty acid translocation and oxidation. In conclusion, FGF-21 is involved in improving ectopic lipid deposition caused by obesity in the liver and skeletal muscles.

## 1. Introduction

Excess triglyceride (TG) in the body to transfer to the liver, skeletal muscle, and other non-adipose tissues in the form of free fatty acids (FFAs) will result in ectopic lipid deposition (ELD) [1]. Ectopic lipid deposition in the liver and skeletal muscle is associated with lipotoxicity, insulin resistance, and metabolic abnormalities [2]. Improving ectopic lipid deposition can alleviate the abnormal lipid metabolism of the liver and skeletal muscle [3]. ELD is easily caused by obesity [4]. Mice fed a high-fat diet exhibit different growth characteristics; that is, some mice could become significantly obese, while other mice weight did not significantly gain [5]. The Lee index is an effective indicator for evaluating the degree of obesity. [6] Mice prone to obesity are defined as diet-induced obesity (DIO), while mice that are difficult to become obese are named diet-induced obesity resistance (DIO-R).

FGF-21 belongs to the latest member of the FGFs (fiber growth factors) family, and 22 members of the family are found in the human body [7]. The biological functions of FGFs are very diverse. So far, studies have found that FGFs participate in regulating a series of physiological activities, including cell differentiation, proliferation, and metabolism [8,9]. Research shows that FGF-21 is expressed in the liver, skeletal muscle, adipose tissue, and other tissues, which can stimulate the oxidation of free fatty acids and the formation of ketone bodies and also inhibit the formation of fat [10,11]. In the liver, the increase in FGF-21 can effectively promote glucose absorption by liver cells in insulin-resistant states [12]. In human skeletal muscle tubes, FGF-21 improves insulin resistance by restoring insulin signaling inhibited by palmitic acid [13].

The characteristic of ectopic lipid deposition is that excessive lipid droplets (LDs) accumulate in non-adipose tissues [14]. LDs primarily contain triglycerides, and their surfaces are covered with monolayer phospholipids inlaid with perilipin (PLINs) [15]. PLIN is the most abundant protein on the lipid droplet surface [16]. It plays an important role in regulating lipid accumulation and regulating the storage of triglycerides [17]. PLINs can be divided into five subtypes according to their structures [18]. PLIN2 and PLIN5 are mainly expressed in the liver and skeletal muscles, which strictly regulate the synthesis and lipolysis of lipid droplets [19,20]. PLIN2 is mainly involved in adipocyte differentiation and promoting the formation and stability of lipid droplets [21]. PLIN5 can recruit mitochondria around lipid droplets, then decompose lipid droplets and reduce lipid toxicity [22]. Fatty acid transferase CD36 (FAT/CD36), located on the cell membrane, plays an important role in the transmembrane transport of free fatty acids [23]. Carnitine palmitoyl transferase-I (CPT-1) is the key rate-limiting enzyme for oxidating free fatty acids, assisting FFA in entering mitochondria, and promoting lipid metabolism [24]. Mechanistically, a high concentration of FFA in serum activates FAT/CD36, which transports more FFA into the cell. Then CPT-1 decomposes FFA under oxidative stress [25,26]. When the oxidative capacity of CPT-1 is exceeded, the excess FFA synthesizes TG and is stored in the cell as lipid droplets, leading to ectopic lipid deposition [27].

In this study, we observed the characteristics of ectopic lipid deposition in the liver and skeletal muscles in mice and evaluated the alteration of FGF-21, perilipin, FAT/CD36, and CPT-1. We aimed to discuss whether FGF-21 is involved in the formation of ectopic lipid deposition in the liver and skeletal muscles and to provide a feasible theoretical basis for future research on improving lipid toxicity.

## 2. Materials and Methods

### 2.1. Animals

C57BL/6J mice (Jinan Peng Yue Experimental Animal Breeding Co., Ltd., Jinan, China) were used to establish the obesity models. All the animal experiments were conducted in compliance with the National Institutes of Health Guide for the Care and Use of Laboratory Animals and approved by the Binzhou Medical University Animal Ethic Committee. Four-week-old mice were randomly divided into a normal group (NC) and a diet-induced obesity group (DIO). The NC group was fed a normal diet, while the DIO group was fed a high-fat diet (Jiangsu Synergy Biotechnology Co., Ltd., Nanjing, China) for 12 weeks. At the end of the 16th week, mice that did not show obesity were selected from high-fat fed mice by calculating the Lee index and defined as obesity-resistant mice (DIO-R). All mice were reared under a 12/12 h light/dark cycle under ventilated, dry conditions at 22 ± 2 °C and with ad libitum access to food and water.

### 2.2. Growth Characteristics

The weight, body length, and body temperature were measured. The body length refers to the distance from the tip of the nose to the anus. The temperature was measured by an infrared thermometer. Lee index passes the following formula: body weight (g) 1/3 × 10^3^/length (cm).

### 2.3. Tissue Sampling

After weighing the experimental mice, the mice were anesthetized by intraperitoneal injection of 4% chloral hydrate at a dose of 1 mL/100 g. The eyeballs of the anesthetized mice were removed, and whole blood was collected and left to stand at room temperature for 20–30 min. Then, centrifuge the whole blood at 3500 rpm for 20 min to obtain the supernatant. The white adipose tissues around the subcutaneous, genitalia, and kidney and the brown adipose tissue under the scapula were separated. Liver and skeletal muscle were separated, and skeletal muscles mainly include the biceps femoris, gastrocnemius, and soleus. They were stored at −80 °C for standby.

### 2.4. Hematoxylin and Eosin (H&E) Staining

The liver and skeletal muscle were fixed with 4% paraformaldehyde (PFA), partially embedded in paraffin, and cut into sections with a thickness of 5 microns. The sections were stained with H&E and then observed under a microscope.

### 2.5. Cell Culture

HepG2 and C2C12 cells were cultured in Dulbecco’s modification of Eagle’s medium (DMEM/High Glucose, HyClone, Logan, UT, USA) supplemented with 10% fetal bovine serum (FBS, Meilunbio, Dalian, China) and 1% penicillin/streptomycin at 37 °C in a humidified atmosphere with 5% CO_2_. When the fusion degree of C2C12 reached 80%, the cells were differentiated with DMEM containing 2% horse serum (Meilunbio, Dalian, China). A large number of mature myotubes could be seen after six days of differentiation. Cells were divided into two groups: the normal control group (NC) and the high lipid group (HL). Cells in the NC group were cultured in serum-free DMEM containing 1% fatty acid-free bovine serum albumin (BSA) alone. Cells in the HL group were incubated in DMEM containing 1% BSA and a 1 mM mixed working solution of palmitic acid and oleic acid for 24 h. A total of 10 mM palmitic acid working solution and 10 mM oleic acid working solution were added into DMEM at a volume ratio of 1:2 to form a 1 mM mixed working solution to induce HepG2 and C2C12 cells to form lipid droplets [28,29].

### 2.6. Cell Transfection

The cells were transfected with a mixture of siRNA-FGF-21 (Gene Pharma, Shanghai, China) and lipofectamine 2000 (Thermo Fisher Scientific, Waltham, MA, USA). Cells are harvested after 48 h. The RNA oligos were designed as follows: negative control, sense:5′UUCUCCGAACGUGUCACGUTT3′, antisense:5′ACGUGACACGUUCGGAGAATT-3′; FGF-21-Homo, sense:5′-GAAGCCGGGAGUUAUUCAATT-3′, antisense:5′-UUGAAUAACUCCCGGCUUCTT-3′; FGF-21-Mus, sense:5′-GAGGACGGUUACAAUGUGUTT-3′, antisense:5′-ACACAUUGUAACCGUCCUCTT-3′.

### 2.7. Enzyme-Linked Immunosorbent Assay (Elisa)

The extraction of cell proteins was repeated, freezing and thawing, by a phosphate-buffered saline (PBS). The BCA kit was used to measure the protein concentration. The expression of carnitine palmitoyl transferase-I (CPT-1) was detected according to the instructions of the ELISA kit (Enzyme-linked Biotech, Shanghai, China).

### 2.8. Oil Red O Staining

The tissues were frozen in liquid nitrogen and cut into 10-micron-thick slices. The slices were stained with an Oil Red O working solution (Titan, Shanghai, China). The cells were first fixed with 4% PFA for 30 min, and then stained at room temperature for 30 min with Oil Red O working solution. The cells were cleaned with PBS and then placed under a microscope for observation.

### 2.9. Western Blot Analysis

The tissues were immersed in an RIPA lysis buffer and centrifuged at 12,000 rpm at 4 °C for 20 min to obtain the supernatant. The total protein concentrations were detected by the BCA assay kit. The protein was concentrated at 80 V and then separated at 120 V. The protein was transferred to a PVDF membrane with a constant current of 200 mA by wet rotation. The membranes were sealed with 5% skimmed milk for 2 h, and then incubated with rabbit anti FGF-21 (Affinity, San Francisco, CA, USA), anti PLIN2 (Proteintech, Wuhan, China), anti PLIN5 (Proteintech, Wuhan, China), and anti GAPDH (Affinity, San Francisco, CA, USA) overnight at 4 °C. We washed the membranes three times with Tris-buffered saline containing Tween-20 (TBST) for ten minutes each time. The membranes were incubated with the peroxidase-conjugated goat anti-rabbit IgG (H + L) at room temperature for 2 h. We washed the membranes three times with TBST for 10 min each time. The membranes were exposed to an ECL super-sensitive luminous solution. ImageJ software (v.1.37; NIH, Bethesda, MD, USA) analyzed the band’s gray value. The cell and tissue experiment methods were consistent.

### 2.10. Statistical Analysis

All values are shown as means ± SD deviation determined using GraphPad Prism software (v.8.0.1, Graphpad Software, San Diego, CA, USA). The data between the two groups were analyzed by an unpaired *t* test. The data of three or more groups were analyzed by a one-way ANOVA. All results were expressed with significant significance as *p* < 0.05.

## 3. Results

1. Some of the mice fed a high-fat diet showed the characteristics to resist obesity.

**Figure 1:**
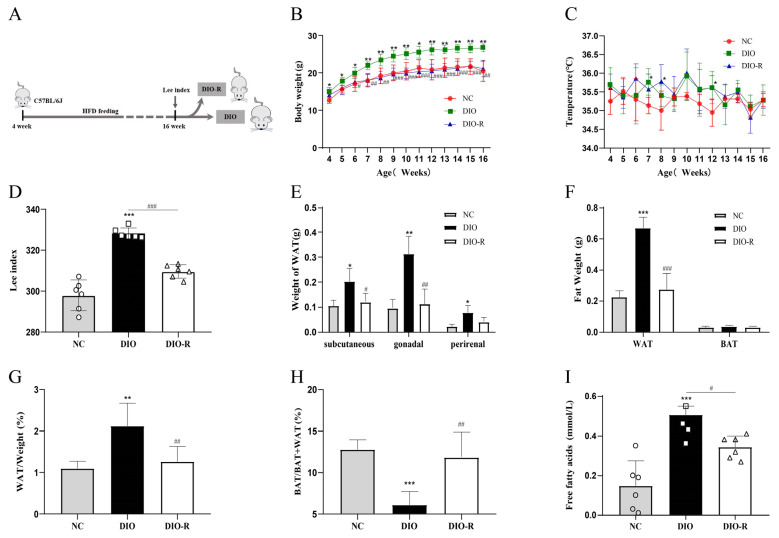
Growth, fat distribution, and biochemical characteristics of obesity-resistance mice. (**A**) Graphic of the animal model design. (**B**) Body weights are presented as the mean ± SD (*n* = 6). (**C**) Body temperatures are presented as the mean ± SD (*n* = 6). (**D**) Lee indexes are presented as the mean ± SD (*n* = 6). (**E**) WAT distribution is presented as the mean ± SD (*n* = 6). (**F**) WAT and BAT weights are presented as the mean ± SD (*n* = 6). (**G**) Body WAT percentages are presented as the mean ± SD (*n* = 6). (**H**) The proportion of the brown fat weight to the total fat weight is presented as the mean ± SD (*n* = 6). (**I**) FFA content in serum are presented as the mean ± SD (*n* = 6). * *p* < 0.05; ** *p* < 0.01; *** *p* < 0.001 vs. the NC group. ^#^ *p* < 0.05; ^##^ *p* < 0.01; ^###^ *p* < 0.001 vs. the DIO group. NC, normal control; DIO, diet-induced obesity; DIO-R, diet-induced obesity resistance.

We have created a timeline to describe the process of establishing an animal model (Figure 1A). All of the mice were sacrificed at 16 weeks, and their tissues were harvested for analysis. After 12 weeks of a continuous high-fat diet, the body weight of the DIO-R group was clearly lower than that of the DIO group (Figure 1B). The body temperatures of the three groups did not differ significantly (Figure 1C). At 16 weeks of age, the Lee index of the DIO group was markedly higher than that in the NC group, and the difference was that the Lee index of the DIO-R group was significantly lower than that of the DIO group (Figure 1D). These data indicate that some mice fed a high-fat diet can resist obesity. Adipose tissue is a major metabolic organ that participates in regulating the dynamic balance of body energy through storing and releasing lipids, playing a leading role in the development of obesity-related metabolic diseases [30]. Therefore, we comparatively analyzed the content of white adipose tissue (WAT) and brown adipose tissue (BAT) in each group. The weight of WAT in different parts, including the subcutaneous, genitalia, and kidneys, of the DIO-R group was lower than that of the DIO group (Figure 1E). The total weight of WAT in the DIO-R group was apparently lower than that in the DIO group (Figure 1F). The ratio of the total WAT weight to body weight in the DIO-R group was significantly lower compared with the DIO group (Figure 1G). The weight of BAT in the three groups had no distinctive difference; however, it can be observed that the proportion of BAT weight to the total fat weight in the DIO-R group was significantly higher than that of the DIO group (Figure 1H). FFA is not only the main source of energy for cells, but it is also a risk factor that can easily induce ectopic lipid deposition [31]. By detecting the content of FFA in the serum, we found that the circulating FFA content in obesity mice was significantly higher than that in normal mice, while the FFA content in the serum of obesity-resistant mice was significantly lower than that of the obesity group (Figure 1I). Overall, some of the mice fed a high-fat diet could resist obesity and perform different growth and biochemical characteristics compared with obesity mice.

2. Obesity-resistant mice exhibited reduced ectopic lipid deposition in the liver and skeletal muscles.

**Figure 2:**
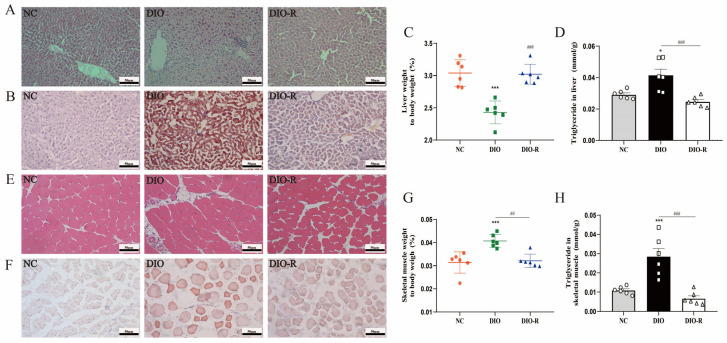
Characteristics of ectopic lipid deposition in the liver and skeletal muscles. (**A**) H&E staining of liver. (**B**) Oil Red O staining of the liver. (**C**) The proportion of liver wet weight to body weight is presented as the mean ± SD (*n* = 6). (**D**) TG content of the liver is presented as the mean ± SD (*n* = 6). (**E**) H&E staining of skeletal muscle. (**F**) Oil Red O staining of skeletal muscle. (**G**) The proportion of skeletal muscle wet weight to body weight is presented as the mean ± SD (*n* = 6). (**H**) The TG content of the liver is presented as the mean ± SD (*n* = 6). Scale bar = 50 μm. * *p* < 0.05; *** *p* < 0.001 vs. the NC group. ^##^ *p* < 0.01; ^###^ *p* < 0.001 vs. the DIO group. NC, normal control; DIO, diet-induced obesity; DIO-R, diet-induced obesity resistance.

To observe the characteristics of ectopic lipid deposition in different groups of mice, we prepared pathology sections in the liver and skeletal muscle. We found by H&E stains that the hepatocytes presented with vacuolar degeneration in the DIO group (Figure 2A). And lots of different-sized lipid droplets were formed in hepatocytes in the DIO group, whereas there were no significant lipid droplets formed in hepatocytes in the DIO-R group (Figure 2B). Moreover, the ratio of liver wet weight to body weight in the DIO-R group was significantly higher than that in the DIO group (Figure 2C). We speculate that this may be because the rate of liver weight loss was much slower than the rate of weight loss. We detected the content of triglycerides and confirmed that the levels of triglyceride in the DIO-R group were significantly lower than that in the DIO group (Figure 2D). These results indicate that obesity-resistant mice exhibit reduced ectopic lipid deposition in the liver. We observed the characteristics of ectopic lipid deposition in skeletal muscles using the same method. Mature adipocytes appeared in the intercellular stroma of skeletal muscle, and lipid droplets formed in the cytoplasm of the skeletal muscle in the DIO group. On the contrary, these phenomena did not emerge in the DIO-R group (Figure 2E,F). The ratio of skeletal muscle weight to body weight declined, and the levels of TG in skeletal muscle were reduced in obesity-resistant mice compared to obesity mice (Figure 2G,H). These data suggest that obesity-resistant mice exhibit reduced ectopic lipid deposition in the skeletal muscle.

3. FGF-21 increased in the liver and skeletal muscle of obesity-resistant mice.

**Figure 3:**
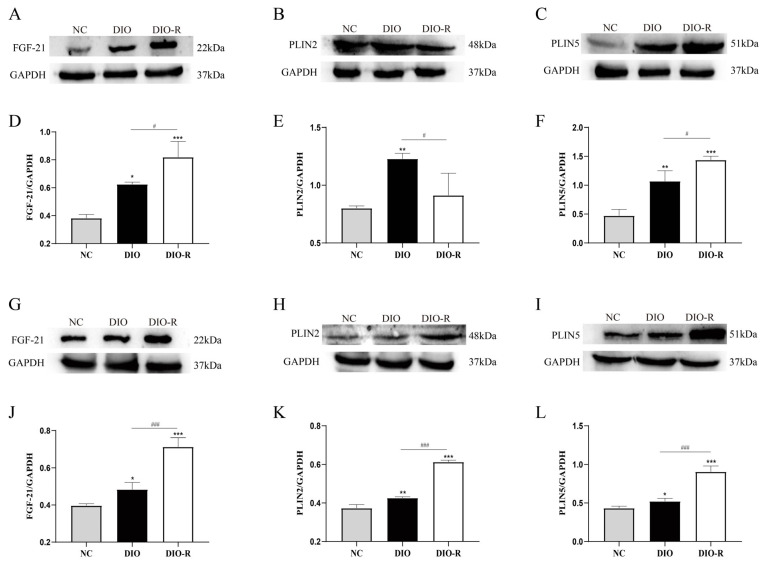
Expression of FGF-21, PLIN2, and PLIN5 in the liver and skeletal muscle. (**A**–**C**) Expression levels of FGF-21, PLIN2, and PLIN5 in the liver by Western blotting (*n* = 3/group). (**D**–**F**) Normalized intensity of FGF-21, PLIN2, and PLIN5 relative to GAPDH, presented as the mean ± SD (*n* = 3/group). (**G**–**I**) Expression levels of FGF-21, PLIN2, and PLIN5 in the skeletal muscle by Western blotting (*n* = 3/group). (**J**–**L**) Normalized intensity of FGF-21, PLIN2, and PLIN5 relative to GAPDH, presented as the mean ± SD (*n* = 3/group). * *p* < 0.05; ** *p* < 0.01; *** *p* < 0.001 vs. the NC group. ^#^ *p* < 0.05; ^###^ *p* < 0.001 vs. the DIO group. NC, normal control; DIO, diet-induced obesity; DIO-R, diet-induced obesity resistance.

Our study already demonstrated that obesity-resistant mice induced by a high-fat diet showed reduced ectopic lipid deposition. Then, we further assessed the expression of FGF-21 and perilipin on ectopic lipid deposition. Compared to the NC group, the protein expression levels of FGF-21 both increased in the DIO group in both the liver and skeletal muscle. Crucially, compared with the DIO group, we found that FGF-21 further increased in the DIO-R group, whether in the liver or skeletal muscle (Figure 3A,D,G,J). The expression of PLIN2 increased in both the liver and skeletal muscle after feeding on a high-fat diet. The levels of PLIN2 in the liver of the DIO-R group were significantly lower than that of the DIO group. On the contrary, the expression of PLIN2 in the skeletal muscle of the DIO-R group was higher than that of the DIO group (Figure 3B,E,H,K). This is because there are no mature adipocytes in the intercellular matrix of the skeletal muscle in obesity-resistant mice, only adipose precursor somatic cells that have not differentiated into mature adipocytes, while PLIN2 is not expressed on the surface of mature adipocytes but only in adipose precursor somatic cells [31]. Different from the expression of PLIN2, we found that the expression of PLIN5 in the DIO-R group was significantly higher than that of the DIO group in the liver and skeletal muscle (Figure 3C,F,I,L). Taken together, our research results reveal that the liver and skeletal muscles of obesity-resistant mice exhibit reduced ectopic lipid deposition with the joint participation of FGF-21 and perilipin.

4. FGF-21 regulated ectopic lipid deposition.

**Figure 4:**
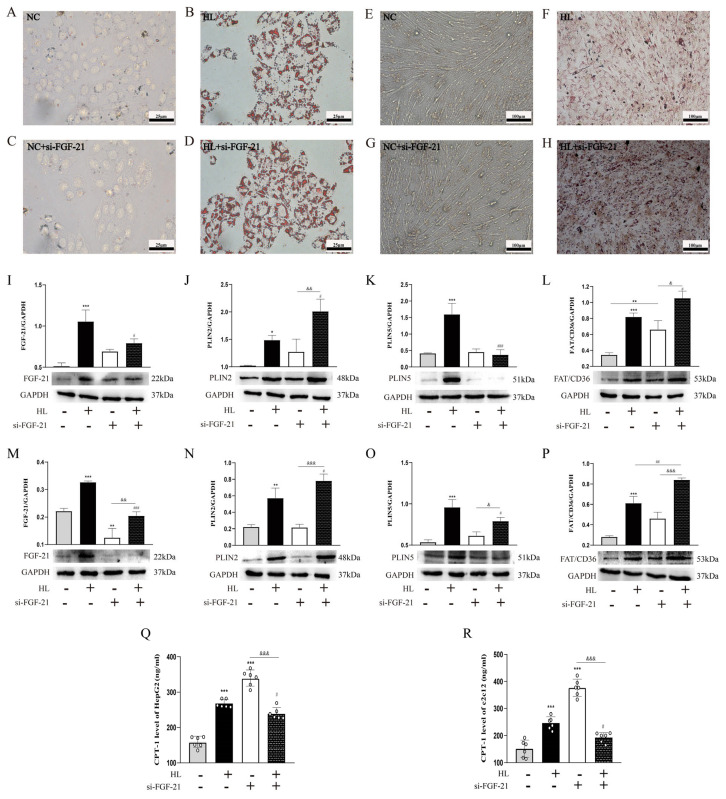
Lipid deposition and related protein expression in HepG2 and C2C12 cells after inhibiting FGF-21. (**A**–**D**) Oil Red O staining of HepG2 cells (scale bar = 50 μm). (**E**–**H**) Oil Red O staining of C2C12 cells (scale bar = 100 μm). (**I**–**L**) Expression levels of FGF-21, PLIN2/PLIN5, and FAT/CD36 in HepG2 by Western blotting, and normalized intensity of FGF-21, PLIN2/PLIN5, and FAT/CD36 relative to GAPDH are presented as the mean ± SD (*n* = 3/group). (**M**–**P**) Expression levels of FGF-21, PLIN2/PLIN5, and FAT/CD36 in C2C12 by Western blotting, and normalized intensity of FGF-21, PLIN2/PLIN5, and FAT/CD36 relative to GAPDH are presented as the mean ± SD (*n* = 3/group). (**Q**,**R**) The levels of CPT-1 in HepG2 and C2C12 are presented as the mean ± SD (*n* = 6). * *p* < 0.05; ** *p* < 0.01; *** *p* < 0.001 vs. the NC group. ^#^ *p* < 0.05; ^##^ *p* < 0.01; ^###^ *p* < 0.001 vs. the HL group. ^&^ *p* < 0.05; ^&&^ *p* < 0.01; ^&&&^ *p* < 0.001 vs. the NC + si-FGF-21 group. NC, normal control; HL, high lipid.

We also established a lipid accumulation model in vitro. As shown in Figure 4A,B,E,F, we observed by Oil Red O staining that lots of different-sized lipid droplets formed in HepG2 and C2C12 cells after a high lipid level was induced. The expression of FGF-21, PLIN2, and PLIN5 increased in HepG2 and C2C12 cells after the stimulation of a high lipid (Figure 4I–K,M–O). Since FAT/CD36 and CPT-1 are involved in free fatty acid translocation and oxidative metabolism, respectively, we also used different methods to detect the expression of these two proteins. A Western blot showed that the expression of FAT/CD36 was significantly increased in the HL group (Figure 4L,P). Using ELISA, we found that CPT-1 also increased after the stimulation of excessive FFAs in the HL group (Figure 4Q,R).

To confirm the relationship between FGF-21 and lipid accumulation, we transfected small interfering RNA into HepG2 and C2C12 cells to inhibit the protein expression of FGF-21. As we expected, lipid droplets increased in HepG2 and C2C12 cells after inhibiting FGF-21 (Figure 4C,D,G,H). Successfully inhibiting FGF-21 markedly upregulated PLIN2 and FAT/CD36 and downregulated PLIN5 and CPT-1 in HepG2 cells after a high lipid was induced (Figure 4I–L,Q). The protein expression trends of PLIN2, PLIN5, FAT/CD36, and CPT-1 after inhibiting FGF-21 in C2C12 cells were the same as those in HepG2 cells (Figure 4M–P,R). These results suggest that inhibiting FGF-21 exacerbates ectopic lipid deposition by affecting perilipin, FAT/CD36, and CPT-1.

## 4. Discussion

In the liver and skeletal muscles, ectopic lipid deposition can accompany insulin resistance and cause lipotoxic damage to target organs, leading to a series of pathological changes such as fatty liver, cirrhosis, and muscle atrophy [32,33]. Therefore, improving ectopic lipid deposition is beneficial for reducing lipid toxicity, reducing abnormal metabolism, and treating obesity-related metabolic diseases. In the previous experiments, we studied the browning of white adipose tissue and found that the subcutaneous white adipose tissue of DIO-R group mice exhibited browning characteristics. Also, the expression of Ucp-1 in the subcutaneous white adipose tissue of DIO-R group mice was significantly higher than that of the DIO group, which can resist obesity by increasing heat production [34]. Our experiment further found that DIO and DIO-R mice show different degrees of differences in energy metabolism, lipid metabolism, and other aspects. The exploration of these differences will help to understand the pathogenesis of metabolic diseases related to obesity. In this study, we found that ectopic lipid deposition in the liver and skeletal muscles of obesity-resistant mice exhibited a reduced and significant increase in FGF-21 expression. And we further elucidate that regulating FGF-21 can improve ectopic lipid deposition by affecting lipid droplet synthesis, decomposition, free fatty acid translocation, and oxidation.

A high-fat diet helps C57BL/6J mice develop obesity, but individuals appear to exhibit varying degrees of susceptibility to obesity [34]. Due to the varying susceptibility of mammals to obesity, individuals exhibit characteristics of obesity or resistance to obesity [35]. To maintain energy homeostasis in the body, obesity-resistant mice showed a reduced intake of a high-fat diet, increased oxidation of visceral fatty acids, and increased energy expenditure after feeding with a high-fat diet [36,37]. Therefore, we suggest that these changes may contribute to the formation of the phenotype of obesity-resistant mice. Our study discovered that after feeding with a high-fat diet, some mice exhibited obese growth characteristics, while others were able to resist obesity (Figure 2A–D). This indicates that, due to differences in body metabolism, mice can exhibit different growth characteristics even under the same feeding conditions. Both the liver and skeletal muscle are prone to ectopic lipid deposition [38]. Through Oil Red O staining and measuring TG content, we found that the positive area of Oil Red O staining in the liver and skeletal muscle of obesity-resistant mice was significantly reduced, and the TG content significantly declined (Figure 3B,D,F,H). These results suggest that the lipid droplet contents in the liver and skeletal muscle of obesity-resistant mice were significantly reduced, and ectopic lipid deposition was significantly reduced. We speculate that the reduction in ectopic lipid deposition in obesity-resistant mice may be regulated by factors related to lipid metabolism. FGF-21 is involved in the pathophysiological processes of multiple metabolic-related diseases [39]. Previous studies have shown that FGF-21 knockout mice exhibit weight gain, increased adipocyte volume, and impaired glucose homeostasis [40]. Increasing the concentration of FGF-21 promotes the activation of brown adipose tissue, the browning of white fat, and glucose metabolism [41]. Our results found that after high-fat feeding, the expression of FGF-21 in the liver and skeletal muscle of obesity mice was compensatory increased compared to normal mice (Figure 4A,D,G,J). However, it is interesting that the expression of FGF-21 in the liver and skeletal muscles of obesity-resistant mice is further elevated compared to obese mice, and the increase in FGF-21 is beneficial for reducing ectopic lipid deposition. Therefore, FGF-21 plays an important role in improving ectopic lipid deposition and may be a potential target for improving ectopic lipid deposition.

Ectopic lipid deposition is often associated with abnormal lipid metabolism, and increased synthesis and weakened decomposition of intracellular lipid droplets are the main causes of ectopic lipid deposition [14]. Perilipin, a key enzyme related to lipid droplet synthesis and decomposition, plays an important role in lipid accumulation [17]. PLIN2 can inhibit lipid breakdown and stabilize lipid storage by limiting the connection between lipase and lipid droplets and can be used for the quantitative analysis of lipid deposition [42]. Related research has shown that the overexpression of PLIN2 in the liver can induce steatosis of the liver [21]. Obesity caused by a high-fat diet also promotes the expression of PLIN2 in skeletal muscles. Consistent with the existing literature reports, in this study, the expression of PLIN2 was significantly increased in the liver and skeletal muscle of obesity mice, while the expression of PLIN2 in the liver of obesity-resistant mice was markedly reduced compared to obesity mice (Figure 4B,E), leading to a decrease in lipid droplet synthesis. However, the expression of PLIN2 in the skeletal muscles of obesity-resistant mice further increased (Figure 4H,K). This may be because there are two types of skeletal muscle ectopic lipid deposition: intramuscular fat and intermuscular fat [43]. In our study, skeletal muscle ectopic lipid deposition was dominated by intermuscular fat (Figure 3E). Intramuscular fat refers to the formation of lipid droplets within skeletal muscle cells, while intermuscular fat refers to the formation of mature adipocytes in the interstitium of skeletal muscle cells [44,45]. The perilipin on the surface of lipid droplets in mature adipocytes is mainly PLIN1, and the perilipin on the surface of lipid droplets in fat precursor somatic cells that do not differentiate into mature adipocytes is mainly PLIN2 [31]. Because mature adipocytes are not formed in the intercellular matrix of the skeletal muscle of obesity-resistant mice, but adipose precursor somatic cells that have not differentiated into mature adipocytes, such as fibroblasts (Figure 3E), the perilipin on the surface of lipid droplets in these adipose precursor somatic cells is mainly PLIN2. Therefore, the expression of PLIN2 in the skeletal muscle of obesity-resistant mice further increased compared to that of obesity mice (Figure 3H,K). In the case of cell lipid overload, PLIN5 can collect mitochondria around lipid droplets, making close physical contact between the two organelles and promoting free fatty acids to flow more effectively from lipid droplets into mitochondria, thus reducing the lipotoxic damage caused by lipid accumulation in cells [22]. Our research results show that the expression of PLIN5 in the liver and skeletal muscle of obesity-resistant mice is higher than that of obesity mice (Figure 3C,F,I,L), indicating that PLIN5 plays an important role in breaking down lipids and reducing lipid accumulation during the formation of ectopic lipid deposition.

This study further explores the potential regulatory effects of FGF-21 on ectopic lipid deposition in the liver and skeletal muscles. After inhibiting FGF-21, we found that lipid deposition further worsened in HepG2 and C2C12 cells (Figure 4A–H). At the same time, the expression of PLIN2 and FAT/CD36 increased in HepG2 and C2C12 cells, while the expression of PLIN5 and CPT-1 decreased (Figure 4I–R). This indicates that inhibiting FGF-21 can exacerbate ectopic lipid deposition by increasing the uptake of free fatty acids, promoting lipid droplet synthesis, reducing lipid breakdown, and declining the oxidation of free fatty acids.

## 5. Conclusions

**Figure 5:**
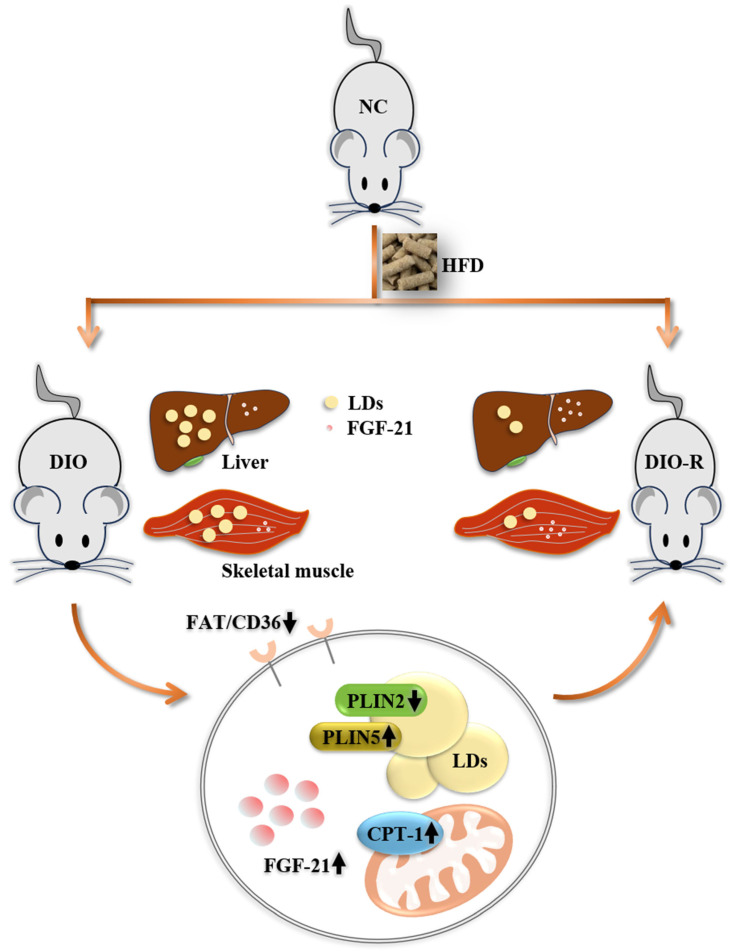
Regulating FGF-21 can improve ectopic lipid deposition by affecting FAT/CD36, PLIN2/PLIN5, and CPT-1.

In summary, this study found that ectopic lipid deposition in the liver and skeletal muscles of obesity-resistant mice was reduced, with a significant increase in FGF-21 expression. In addition, we reveal that FGF-21 can improve ectopic lipid deposition by regulating lipid droplet synthesis and decomposition, as well as free fatty acid translocation and oxidation (Figure 5). Our research contributes to feasible targets for improving ectopic lipid deposition and treating obesity-related metabolic diseases.

## Data Availability

The data presented in this study are available in this article.
